# Persuasive Technology in an mHealth App Designed for Pelvic Floor Muscle Training Among Women: Systematic Review

**DOI:** 10.2196/28751

**Published:** 2022-03-22

**Authors:** Aida Jaffar, Chai-Eng Tan, Sherina Mohd-Sidik, Novia Admodisastro, Felicity Goodyear-Smith

**Affiliations:** 1 Department of Psychiatry Faculty of Medicine and Health Sciences Universiti Putra Malaysia Selangor Malaysia; 2 Primary Care Unit Faculty of Medicine and Defence Health Universiti Pertahanan Nasional Malaysia Kuala Lumpur Malaysia; 3 Department of Family Medicine Faculty of Medicine Universiti Kebangsaan Malaysia Kuala Lumpur Malaysia; 4 Software Engineering & Information System Department Faculty of Computer Science & Information Technology Universiti Putra Malaysia Selangor Malaysia; 5 Department of General Practice and Primary Health Care University of Auckland Auckland New Zealand

**Keywords:** urinary incontinence, pelvic floor muscle training, mHealth app, persuasive technology, capability, opportunity, and motivation–behavior model, mobile phone

## Abstract

**Background:**

Pelvic floor muscle training (PFMT) is one of the first-line treatments for stress urinary incontinence among pregnant women. Mobile health (mHealth) technology is potentially effective for delivering PFMT to pregnant women. Persuasive technology in the development of such mobile apps may facilitate behavior change by improving adherence to the exercises. The Capability, Opportunity, and Motivation–Behavior (COM-B) model is potentially useful in selecting the appropriate interventions to be incorporated into the apps.

**Objective:**

This review of mHealth apps for PFMT aims to describe the principles of persuasion used for each app and to propose mHealth app design features based on the COM-B model.

**Methods:**

A systematic literature search was conducted to answer three main research questions: what are the available mHealth apps for PFMT in the published literature, what persuasive strategies were used in their studies how were they mapped to the COM-B model, and how effective were the selected persuasive strategies for PFMT adherence? We searched PubMed, CINAHL, Web of Science, Scopus, and local Malaysian databases such as MyCite and MyMedR for articles reporting mHealth apps used for the delivery of PFMT. We included original articles reporting experimental and cross-sectional studies, including pilot or feasibility trials. Systematic and narrative reviews were excluded. Narrative and thematic syntheses were conducted on the eligible articles based on the research questions. The Cochrane risk of bias tool and the Risk of Bias Assessment Tool for Non-randomized Studies were used to assess study bias.

**Results:**

Of the 169 records from the initial search, 10 (5.9%) articles meeting the selection criteria were included in this review. There were 8 mHealth apps designed for the delivery of PFMT. The Tät, which used 3 categories of persuasive system design, improved PFMT adherence and was cost-effective. Only 1 app, the iBall app, used all categories of persuasive system design, by including social support such as "competition" in its design. The Diário Saúde app was the only app developed using operant conditioning. All apps incorporated Tailoring and Expertise as part of their PSD strategies. Only 3 apps, the Diário Saúde, Tät, and Pen Yi Kang demonstrated improved PFMT adherence.

**Conclusions:**

Persuasive technology used in mobile apps may target desired behavior change more effectively. The persuasive system design can be mapped to the COM-B model to explain its effectiveness on behaviour change outcomes.

## Introduction

### Background

Urinary incontinence (UI) is defined as involuntary urinary leakage or inability to control urine. Various physiological changes during pregnancy, including collagen changes, hormonal changes, and increased uterine and fetal weight, contribute to the weakening of the pelvic floor muscles (PFMs) during pregnancy [1]. Approximately 42% of women experience their first UI during pregnancy, and up to 31% of parous women have UI [2]. Women with persistent UI after delivery may continue to experience UI for another 12 years [3]. Therefore, UI during pregnancy may be an essential risk factor for subsequent UI among women.

UI is troublesome, particularly during pregnancy, and affects women’s quality of life physically, emotionally, spiritually, and financially [4-6]. Those who experience it may resort to various methods to deal with the problem, including using pads or incontinence diapers and avoiding social situations because of embarrassment. However, many pregnant women do not seek help despite having UI symptoms because of the perception of UI as a normal pregnancy change or embarrassment to initiate a discussion about UI with their health care provider [7,8]. Some are unaware of treatment availability, such as PFM training (PFMT), whereas others feel they should not disturb their health care provider as UI is a temporary issue [4].

PFMT is a repetitive, voluntary contraction and relaxation of specific PFMs that is recommended for managing and preventing UI during pregnancy and after delivery [9,10]. Focused PFMT is useful for strengthening the PFMs and reducing UI [11]. PFMT is low-cost and noninvasive and has benefits for the prevention and treatment of UI among pregnant women [9].

However, PFMT requires adherence and correct technique to work. Lack of knowledge and skills for PFMT is a barrier to successful management of UI [12,13]. Women report lack of self-confidence, difficulty remembering, and time constraints [7,14-16] as barriers to performing PFMT. This results in the underuse of PFMT by those who experience UI.

Antenatal health care providers need training on how to teach PFMT, and some have difficulties in allocating time for teaching PFMT to pregnant women [7]. In addition, no standard national guidelines for PFMT are available to guide health care providers regarding the appropriate frequency of PFMT and other technical details [4,7]. Therefore, efforts to develop mobile health (mHealth) apps to deliver PFMT have been made to address these challenges.

mHealth is defined as “the use of wireless communication devices to support public health and clinical practice” [17]. mHealth apps are software apps used by health care professionals and patients for conveying health knowledge, research to improve health treatments, and public health [18]. mHealth apps can be classified based on the target users: health care professionals and patients. mHealth apps for patients can be further divided into five subcategories: (1) educational health apps, (2) apps to contact health care professionals, (3) apps to check personal health records, (4) personal care apps, and (5) social networking apps [18].

mHealth apps for PFMT are personal care apps that assist users in training their PFMs, akin to a personal trainer. The apps aim to modify negative attitudes among users to successfully produce behavior change. Developing a positive attitude is essential to enable people to change their behavior successfully [19]. The use of persuasive technology (PT) in mHealth apps may support attitude and behavior change in users to adopt PFMT as part of their lifestyle.

### PT in App Design

PT is defined as “interactive computing systems designed to aid and motivate people to alter their attitude and behaviours” [20]. PT can be categorized according to its functional roles, which are tools, media, or social actors [20]. Tools help users perform a target behavior by making the task easier or restructuring the task. Media represents the content that supports users in repeating a behavior or providing emotional support that facilitates the target behavior. Social actors are cues for social responses that promote the target behavior. These functions can be incorporated into the design of an mHealth app for behavior change.

Oinas-Kukkonan [21] defined persuasive systems as “computerized software or information systems designed to reinforce, change or shape attitudes or behaviors or both without using coercion or deception.” Adopting a persuasive system design (PSD) may provide effective persuasion [22]. There are three potential successful outcomes for a persuasive system: voluntary reinforcement and change or shaping of attitudes or behaviors. PSD for PFMT apps can be divided into four main categories—primary task support, dialogue support, credibility support, and social support—as seen in Table 1.

App designers need to translate theoretical determinants of behavior into technology design items [23]. Identifying suitable PT elements for the design of PFMT mHealth apps should suit the context of pregnant women who wish to adopt PFMT and the evolving physiological changes of pregnancy.

**Table 1:** Persuasive system design (PSD) and suggestions for the pelvic floor muscle training (PFMT) apps.

PSD category and subcategories		Suggestions for PFMT app features
**Primary task support**		
	Reduction	The app lists effective intervention for UI^a^.
	Tailoring	The app provides PFMT information according to the target user characteristics.
	Personalization	The app provides personalized content according to the individual user.
	Self-monitoring	Users are able to monitor their progress.
**Dialogue support**		
	Rewards	A trophy is given after the user has completed PFMT schedule.
	Reminders	This is a crucial feature because PFMT needs to be done daily.
	Liking	Likable minimalist design with user’s choice of colors
	Social role	Use of a virtual physiotherapist
**Credibility support**		
	Trustworthiness	The app provides unbiased information regarding PFMT.
	Expertise	The app provides the professional background and expertise of the content developers.
	Surface credibility	The app appears professional.
	Authority	The app bears the logo of the developer’s institution.
	Third-party endorsements	The app includes endorsing statements from relevant professionals such as physiotherapists or urogynecologists.
**Social support**		
	Social learning	The app allows users to see the deidentified general performance of all users.
	Social comparison	The app also allows users to share their achievements with other users.
	Normative influence	The app normalizes the experience of UI and learning PFMT by connecting a user with other similar users.

^a^UI: urinary incontinence.

### The Capability, Opportunity, and Motivation–Behavior Model

The Capability, Opportunity, and Motivation–Behavior (COM-B) model has great potential as a theory to guide the selection of persuasive design elements for app development [24]. It is a simple, validated model derived from combining various behavior change theories.

The *Capability* domain in COM-B proposes that a person must acquire correct knowledge, skills, and abilities to allow them to perform a targeted behavior. In the context of pregnant women and PFMT, capability can be classified into two subcategories: (1) physical capability (referring to the woman’s physical skills) and (2) psychological capability (referring to the woman’s understanding of PFMT and ability to remember to practice PFMT). Both capabilities are required to affect behavior change. Pregnant women in primary care settings lack the necessary knowledge about PFMT, whereas those with good PFMT knowledge may still have poor practices as a result of poor physical capability [12]. Hence, improving PFMT knowledge and skills is a crucial aspect of intervention, which can be addressed through training such as via an educational video or a group intervention session with a physiotherapist.

The *Opportunity* domain in COM-B refers to external factors that influence the targeted behavior. Again, in the context of pregnant women and PFMT, opportunity may consist of (1) physical opportunity (availability of environmental, nonliving elements that support PFMT, such as a private location to learn and perform PFMT) and (2) social opportunity (related to the opportunities afforded by people around the pregnant women, eg, cultural and social norms related to PFMT). The opportunity to learn PFMT in their own comfort and privacy is a form of physical opportunity that can be created by mHealth apps. Having health care providers promote the use of apps to learn PFMT can be a form of social opportunity. Incorporating a function in which users can post questions or communicate with experts regarding UI improves accessibility to knowledge and may also represent the creation of social opportunity for pregnant women [25].

The *Motivation* domain refers to an internal process that influences the decision to change and adopt the targeted behavior. Motivation is divided into (1) reflective motivation (referring to reflective thought processes that improve motivation, eg, identifying solutions to schedule PFMT thrice daily in their daily activities) and (2) automatic motivation (emotional or habitual processes that improve motivation, eg, reinforcing their PFMT routine through reminders and rewards).

The development of effective evidence-based behavioral interventions should incorporate suitable health behavior theories with qualitative and quantitative evidence [26,27]. Previous PFMT interventions have been designed based on various health behavior theories such as the social cognitive theory and the theory of planned behavior [28]. Social cognitive theory is limited by its focus on social influences and self-efficacy, whereas the theory of planned behavior focuses on motivation for PFMT adherence. Therefore, a more comprehensive behavior change theoretical framework that encompasses major theories is desirable [24,29]. The strength of the COM-B model lies in its simplicity in identifying the key components that affect a person’s motivation and behavior.

The aim of this review is to (1) list the mHealth apps designed for PFMT, (2) determine the PSD used, and (3) suggest PSD features for new mHealth apps that incorporate the COM-B model.

## Methods

### Overview

We conducted a systematic literature search for published articles on mHealth apps for PFMT among women in December 2020. For the purpose of this paper, *mHealth app* is defined as an app developed for delivery via a mobile device, including a tablet device, for health care purposes [30]. This definition excludes email, websites, telemedicine, and telehealth.

This literature search aimed to answer three main research questions: (1) What are the available mHealth apps for PFMT in the published literature? (2) How effective were the selected persuasive strategies for PFMT adherence? and (3) What persuasive strategies were used in their studies and how did they map to the COM-B model?

The search was conducted in the PubMed, CINAHL, Web of Science, and Scopus databases, which are reference databases in biomedicine and rehabilitation and comprise the largest general scientific databases [31]. We also searched the Malaysian databases MyCite and MyMedR using the following search keywords: *(pelvic floor muscle training OR pelvic floor muscle exercise OR Kegel exercise) AND (women) AND (digital health OR mhealth OR mobile health OR mobile application OR smartphone OR mobile app OR smartphone app).* The initial intent of this review was to look at the persuasive strategies used by mobile apps for PFMT in pregnant women. However, because of the extremely limited number of published articles specifically on pregnant women, we expanded the population to include women in general.

### Selection Criteria

The selection criteria for this review are listed in Textbox 1.

Titles, abstracts, and full-text articles were further assessed and discussed by 2 reviewers (AJ and CET). Consensus for article inclusion was achieved through discussion with a third reviewer (SMS). Further discussion focusing on persuasion was undertaken with NA, who is the expert in the area. In addition, a manual search was conducted using reference lists of selected articles to identify any additional suitable records that were not included in the database search results.

**Selection criteria for the literature search.**

**Inclusion criteria**

- Population: mobile health (mHealth) apps for pelvic floor muscle training (PFMT) for women
- Intervention: any mHealth app intervention in an educational or training context for patients or a targeted population that enables improvement in knowledge, self-efficacy, and adherence. All types of mHealth apps (eg, synchronous [connected with the health care providers] or asynchronous [stand-alone]) will be included.
- Comparison: usual care
- Outcome: PFMT adherence and usability
- Type of study design: all types of randomized controlled trials, cross-sectional studies, case–control studies, and feasibility studies
- Publication type: published primary or secondary data in a peer-reviewed journal
- Language: English and Malay
- Publication year: only articles published between 2010 and 2020. A preliminary search conducted before this review found that most of the articles regarding PFMT mHealth apps were published after 2010.

**Exclusion criteria**

- Population: mixed users (men and women) of mHealth apps for urinary incontinence education or other pelvic disorders without PFMT information
- Intervention: reminder system via email, SMS text message, or WhatsApp
- Type of study design: study protocol and validation study
- Publication type: reviews, systematic reviews, conference proceedings, abstract only, book chapter reviews, letters, and editorials
- Language: other than English and Malay

### Data Extraction

Both reviewers extracted data from eligible articles and entered them into a Microsoft Excel spreadsheet, including data on study characteristics (year of publication, design, sample size, and duration of follow-up), participant characteristics (sex, age, and type of illness), the intervention (persuasive strategies, behavior change theories, features of the technology, and biofeedback), and results (outcomes of PFMT and adherence). Both reviewers compared and discussed the extracted results to achieve a consensus. The third reviewer (SMS) was called to discuss in the event of any unresolved discrepancies.

### Data Synthesis

We conducted a narrative synthesis to answer the first 2 research questions, which aimed to list available apps for PFMT among women and their effectiveness. A full meta-analysis was not feasible owing to the heterogeneity of the studies and insufficient studies with an experimental design. A thematic synthesis was conducted to answer the third research question, which was to determine the persuasive design strategies used by the apps. The findings were then mapped to the COM-B model.

### Quality Appraisal

The quality of eligible randomized controlled trials was assessed by AJ and CET using the Cochrane risk of bias tool, concentrating on sequence generation, allocation concealment, blinding, attrition bias, completeness of outcome data, and other sources of bias [32]. For nonrandomized studies, the Risk of Bias Assessment Tool for Non-randomized Studies was used, which has demonstrated its validity to assess the bias specific to observational studies [33]. Any conflicts were resolved through discussion with a third person.

## Results

### Search Results and Study Characteristics

**Figure 1:**
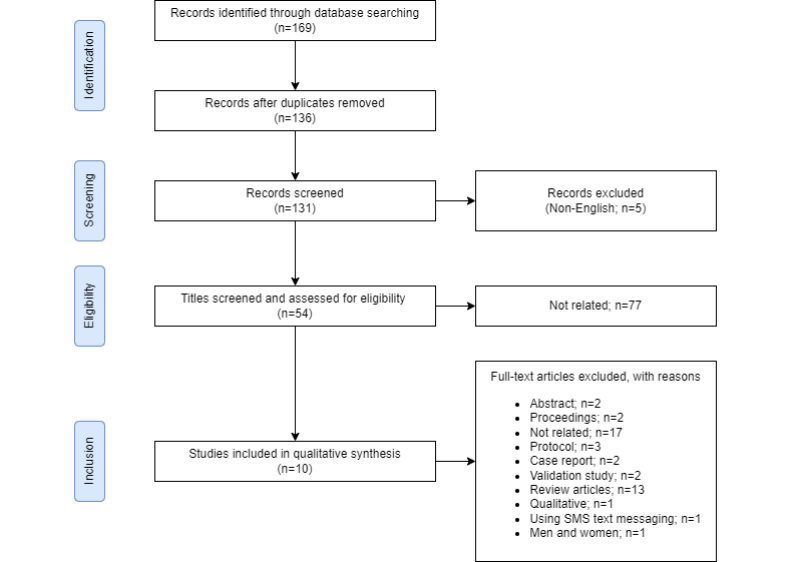
PRISMA (Preferred Reporting Items for Systematic Reviews and Meta-Analyses) flow diagram.

From the 169 records screened for titles and abstracts, 54 (32%) full texts were reviewed, and 10 (5.9%) articles met our selection criteria and were included in the review (Figure 1) [34-43]. These 10 articles were published between 2015 and 2020. Of the 10 articles, 3 (30%) referred to the same app, Tät [35,39,42].

### Available mHealth Apps for PFMT

There was a total of 8 mHealth apps developed for PFMT (Table 2). Of the 10 studies, 3 (30%) investigated the effectiveness of stand-alone apps [35,39,42], and 1 (10%) used an audio guidance app [43]. Another study (1/10, 10%) evaluated an app that uses Bluetooth technology to link data from biofeedback [36]. Of the 8 apps, except for 1 (13%) [40], all mHealth apps (n=7, 88%) provided asynchronous PFMT—there was no live communication with a trainer.

**Table 2:** Details of the primary studies identified and reviewed.

PFMT^a^ mHealth^b^ app	Country	Year launched	Platform	Study design	Study participants	Participant characteristics	Outcomes
Squeezy App [41]	United Kingdom	2013	iOS	Cross-sectional survey	464—38% pregnant women; mean age unavailable	Mixture of bladder problem and healthy men and women	80% carried out their PFMT at least 3 days a week.
Tät [35]	Sweden	2013	iOS and Android	Randomized controlled trial	123 nonpregnant women; mean age (intervention group): 44.8 (SD 9.7) years; mean age (control group): 44.7 (SD 9.1) years	Stress urinary incontinence	41% (25/61) performed PFMT daily
Tät [39]	Sweden	2013	iOS and Android	Randomized controlled trial (2-year follow-up)	123 nonpregnant women; mean age: 44.2 (SD 10.3) years	Stress urinary incontinence	Use of incontinence protection decreased significantly (*P*=.04); 31/46 (67.4%; *P*<.001) could contract their pelvic muscles correctly
Tät [42]	Sweden	2013	iOS and Android	Randomized controlled trial (cost-utility study)	123 nonpregnant women; mean age (intervention group): 44.8 (SD 9.7) years; mean age (control group): 44.7 (SD 9.1) years	Stress urinary incontinence	The extra cost per quality-adjusted life year for the app group ranged from −€2425.70 (US $2718.60) to €14,870.60 (US $16,666.20)
Diário Saúde [34]	Brazil	2016	N/A^c^	Randomized controlled trial	31 nonpregnant women; mean age (intervention group): 47.2 (SD 10.6) years; mean age (control group): 53.3 (SD 13.2) years	Stress urinary incontinence	Adherence was higher in the app group at 1 and 2 months after PFMT (*P*<.001)
Penyikang app [40] (synchronous app)	China	2017	iOS and Android	Cross-sectional study	1982 postpartum women aged >18 years	Pelvic floor muscle weakness	483 postpartum women had a relatively high degree of participation (15 times)
iBall app [36] (an external device connected via Bluetooth)	Canada	2017	N/A	Pilot randomized controlled feasibility study	23 postpartum women; mean age (intervention group): 31 (SD 2.7) years; mean age (control group): 34 (SD 2.2) years	Not specified	There was no statistically significant difference between the groups for change scores
MyHealtheBladder app [37]	United States	2017	N/A	Pilot single-group, quasi-experimental study	29 nonpregnant women; mean age: 54.4 (SD 10.4) years	Urinary incontinence	97% adherence rate to the daily sessions
Bwom app [38]	United States	2017	N/A	Cross-sectional survey	47 patients and 22 providers (pregnancy status not available); age 20-50 years	Not specified	No adherence outcome
Pen Yi Kang [43] (audio guidance app)	China	2018	iOS and Android	2-arm parallel randomized controlled clinical trial	108 primipara women; mean age (intervention group): 29.2 (SD 2.6) years; mean age (control group): 29.1 (SD 2.9) years	Stress urinary incontinence	Greater self-efficacy with a mean difference of 8.9 points at 6 months after delivery

^a^PFMT: pelvic floor muscle training.

^b^mHealth: mobile health.

^c^N/A: not applicable.

### Study Quality Assessment Results

For randomized controlled trials, the detailed quality assessment results are summarized in Figure S1, Multimedia Appendix 1 [34-43]. In general, all trials (4/4, 100%) included in this review showed an acceptable risk of bias. The randomization sequence was adequately generated in 50% (2/4) of the trials, and all trials (4/4, 100%) adequately concealed the allocation of the participants. Blinding of the participants and personnel was not possible in these trials. Therefore, the risk of performance bias in all studies was classified as low. All articles reported an intention-to-treat analysis except for Araujo et al [34], who conducted a per-protocol analysis.

For nonrandomized studies, the detailed results of the quality assessment are summarized in Figure S2, Multimedia Appendix 1. In general, none of the 4 studies reported a strategy for minimizing selection bias and controlling methods for the confounders. More than half of the studies (3/4, 75%) had low response rates (<50%), and only 25% (1/4) of the studies reported the point estimates of the results.

### Persuasive Strategies and COM-B Model in the PFMT Apps

Only the iBall app (1/8, 13%) used all 4 categories of PSD (Table 3) [36].

**Table 3:** Details of the primary studies identified with their persuasive system design (PSD) categories.

PFMT^a^ mHealth^b^ app	PSD category			
	Primary task	Dialogue support	Credibility support	Social support
Squeezy App [41]	Tailoring, personalization, and self-monitoring	Reminders	Expertise	N/A^c^
Tät [35]	Tailoring, tunneling, and self-monitoring	Reminders	Expertise	N/A
iBall app (an external device connected via Bluetooth) [35]	Tailoring, personalization, and self-monitoring	Rewards	Expertise	Competition
Bwom app [38]	Tailoring, personalization, and self-monitoring	Social role	Trustworthiness, expertise, surface credibility, and third-party endorsements	N/A
Diário Saúde^d^ [34]	Tailoring, personalization, and self-monitoring	Reminders	Expertise	N/A
Penyikang app [40]	Tailoring	N/A	Expertise	N/A
MyHealtheBladder app [37]	Tailoring, personalization, and self-monitoring	Rewards and reminders	Expertise	N/A
Pen Yi Kang [43]	Tailoring, personalization, and self-monitoring	Reminders	Expertise	N/A

^a^PFMT: pelvic floor muscle training.

^b^mHealth: mobile health.

^c^N/A: not applicable.

^d^Operant conditioning.

Of the 8 mHealth apps to improve PFMT adherence, only the Diário Saúde app (n=1, 13%) [34] was developed using operant conditioning. Operant conditioning occurs by providing reinforcement to a certain behavior immediately when the behavior is performed [44]. This helps increase the likelihood that the behavior will be performed again by the user. In the intervention involving the Diário Saúde app, the study participants underwent surface electromyography (sEMG) and PFM examination by a single physiotherapist during PFMT. All the study participants were given immediate feedback on whether they had performed PFMT correctly and were able to observe the sEMG feedback for themselves. Their sEMG graph was then displayed in the app to assist their PFMT at home [34]. This represented an incentive or reinforcement for correctly performing PFMT with the aid of the app.

All the apps (8/8, 100%) incorporated *Tailoring* (under the *Primary Task* category) and *Expertise* (under the *System Credibility* category) as part of their PSD strategies. *Tailoring* is defined as “any of a number of methods for creating communications individualized for their receivers, with the expectation that this individualization will lead to larger intended effects of these communications” [45]. All the apps (8/8, 100%) tailored the intervention by matching the content to the user groups. For *Expertise*, the apps portrayed the credibility and competence of their content by highlighting the expertise of educators in the field of PFMT, such as physiotherapists or physicians.

Although the Bwom app [38] incorporated the most persuasive strategies from the *System Credibility* category, the study did not report the effectiveness of the app. Instead, it was a cross-sectional study aimed at surveying the usefulness of the app for PFMT. The assessment showed that both the understandability and actionability of the app were >90%. These high scores may support the value of the app design among women with incontinence.

All the apps (8/8, 100%) had persuasive strategies that could be mapped to the *Opportunity* and *Motivation* domains of the COM-B model to influence and improve the behavior, which is PFMT (Table 4). Most apps (7/8, 88%) also allowed users to perform self-monitoring. This could be mapped to the *Capability* domain of the COM-B model.

**Table 4:** Details of the primary studies identified with the Capability, Opportunity, Motivation–Behavior (COM-B) model domains.

PFMT^a^ mHealth^b^ app	COM-B model							
	Capability			Opportunity			Motivation	
	Psychological	Physical	Physical		Social	Reflexive		Automatic
Squeezy App [41]	Information and links	Visual aid	Overall design of the app (look and feel)		Not available	Snooze		Reminders
Tät [35]	Information on the pelvic floor, stress UI^c^, and lifestyle	PFMT skill training	Ability to use the app		Not available	Statistic function and goals		Reminders
iBall app [36] (an external device connected via Bluetooth)	Not available	PFMT skills via gamification	N/A^d^		Gamification—ranking score (web community for original version)	Not available		Rewards
Bwom app [38]	Educational videos on PFMT	Provides list of PFMT exercise plans	Ability to use the app (PEMAT^e^)		Culturally relevant	Not available		Not available
Diário Saúde [34]	Not available	The visual component of sEMG^f^	Ability to use the app		Not available	Statistic function and goals		Reminders
Penyikang app [40]	Facilitate PFD^g^ information	Self-management	Participation using the app		Ability to consult with the physicians (synchronous app)	Not available		N/A
MyHealtheBladder app [37] (web-based information)	A story‐based behavioral program	PFMT strategies	Ability to use the app		N/A	A story-based behavioral program		Rewards and reminders
Pen Yi Kang [43]	N/A	PFMT guidance—audio	Ability to use the app		N/A	N/A		Reminders

^a^PFMT: pelvic floor muscle training.

^b^mHealth: mobile health.

^c^UI: urinary incontinence.

^d^N/A: not applicable.

^e^PEMAT: Patient Education Materials Assessment Tool.

^f^sEMG: surface electromyography.

^g^PFD: pelvic floor dysfunction.

### The Effect of the Selected Persuasive Strategies

Diário Saúde, Tät, and Pen Yi Kang [34,35,43] demonstrated improved PFMT adherence. For Tät, 41% of the participants performed PFMT daily, and 42.6% performed PFMT weekly after 12 weeks of using the app [35]. For Pen Yi Kang, self-efficacy for adherence to PFMT was measured as its outcome. The app resulted in a mean difference of 8.9 points for self-efficacy at 6 months after delivery when compared with the control group (conventional home-based PFMT) [43]. This suggests that the audio guidance was beneficial for improving users’ self-efficacy and maintaining PFMT adherence. Hence, the incorporation of persuasive strategies in mHealth apps resulted in an improvement in PFMT adherence.

PFMT adherence among users of the MyHealtheBladder [37] and Squeezy apps [41] was good. However, the adherence to these apps was determined using cross-sectional or quasi-experimental study designs. Therefore, the effectiveness of the apps on adherence needs to be interpreted with caution as there was no control group for an objective comparison.

Meanwhile, the iBall app was unable to demonstrate improved adherence owing to technical difficulties and discomfort faced by study participants because of the external device [36]. Although the iBall app had various sophisticated persuasive features such as an interesting user interface, Bluetooth connection with an external device, and gamification, these features were not sufficient to overcome the problems with user acceptance of the external device. Thus, the potential effectiveness of the app itself in supporting adherence was hampered by the low acceptability of the external device.

## Discussion

### Persuasive Design in mHealth Apps

The search found 8 eligible PFMT mHealth apps with mixed findings. Diário de Saúde, which used operant conditioning, showed a significant improvement in adherence rate [34]. *Expertise* and *Authority* were the most commonly chosen persuasive strategies in mHealth apps for PFMT.

Persuasive strategies are essential for attracting the target users’ attention or interest in the app. The background of health care professionals and researchers who recommend the app and its advantages to target users is important in attracting their interest. This approach was explained in detail by Squeezy App as follows: “...has been curated by a specialist women’s health physiotherapist, peer-reviewed and endorsed by the National Health Service (NHS)” [41]. This statement was important to convince users that the app was a genuine product developed from trusted sources and compliant with the Data Protection Act [41].

Recommendation by a specialist or expert provides a sense of trust for potential users regarding the importance and safety of the app. This motivates users to continue using the app, improve their adherence, and gradually form habits for the desired target behavior. Squeezy App was designed to be used with or without physiotherapist guidance. Therefore, the credibility of the app is important as users may not have the opportunity to meet the physiotherapist in person for PFMT. Evaluation of the app by physiotherapists and their endorsement of the app increased users’ confidence in it [41].

Operant conditioning, which is an important concept in behavior change theories, was used by the Diário de Saúde app [34]. Displaying the biofeedback chart through the app provided users with feedback that reinforced their PFMT exercises [34]. This illustrates a successful use of behavior change theories to improve adherence to PFMT. The Tät app has been recently proven effective in reducing incontinence for urge UI and mixed UI after a 15-week intervention [46] and a 12-week intervention for UI self-management based on a prospective cohort study [47]. However, most studies did not include pregnant women. More studies are needed to determine the effectiveness of mHealth solutions for pregnant women who experience urinary incontinence. A future study has been planned to determine a mobile app’s effectiveness in reducing incontinence among pregnant women [48].

### Applying the COM-B Model

The COM-B model can be applied to address barriers to PFMT and identify potential behavior change techniques or persuasive strategies to be incorporated into mHealth app design. For example, barriers to PFMT may include being unaware of the need to perform PFMT, inability to understand how to perform PFMT, and lack of self-efficacy [4,7,12,14]. Performing PFMT can be challenging as the PFMs are not visible [13]. Thus, health care providers need to explain clearly and guide pregnant women to visualize the contraction of the correct muscles [7]. This represents the creation of psychological capability in the COM-B model. Therefore, an app that provides training with gradually increasing levels of difficulty would be helpful in improving the psychological capability of users. The reminder function in the app also assists users in remembering when to perform the behavior. The ability to self-monitor their performance, including the provision of rewards for achieving continuous adherence to PFMT, is a persuasive strategy that could provide additional support for the psychological capability component of the COM-B model.

Other barriers to PFMT include limited access to PFMT information and lack of time [4,13]. Providing PFMT information through mHealth apps that can be accessed at one’s own convenience is a form of physical opportunity in the COM-B model. Social barriers to PFMT may include a lack of positive role models for performing PFMT, embarrassment, and the social norms among friends and colleagues that hinder a person from performing PFMT [4,13]. Creating a community of app users (eg, an app that includes a Kegel exercise support group) may provide the social opportunity to keep the users motivated to perform PFMT.

Motivating a person to change their behavior is challenging. Reflective motivation based on the COM-B model may be helpful. This may be in the form of guiding app users to evaluate and plan for specific behavior, such as setting the time for PFMT. Consciously reflecting on personal barriers to performing the target behavior and solving them can help motivate a person. This could be incorporated into apps through guided reflections.

This review was limited to mHealth for delivering PFMT to women. There may be other PSD and behavior change theories used in other PFMT apps designed for men. The focus of this review was only on the COM-B model. Other widely used theories, such as the Health Belief Model and Social Cognitive Theory were not discussed in this review.

### Conclusions

There have been 8 mHealth apps designed for PFMT among women worldwide in the past decade. Physical capability can be improved by using PFMT skill training and system credibility (expertise) as persuasive strategies. Physical opportunity for PFMT is supported by the app’s usability (personalization), whereas the users’ motivation can be improved using goals and reminders. The persuasive strategies in these apps were mainly mapped to the *Capability* and *Motivation* components of the COM-B model. Tailoring and self-monitoring were the most commonly used persuasive strategies in the design of PFMT apps.

## Appendix

Multimedia Appendix 1 Quality assessment of the included studies (randomized and nonrandomized controlled trials).

## Abbreviations

COM-B: Capability, Opportunity, and Motivation–Behavior
mHealth: mobile health
PFM: pelvic floor muscle
PFMT: pelvic floor muscle training
PSD: persuasive system design
PT: persuasive technology
sEMG: surface electromyography
UI: urinary incontinence

## References

[1] Sangsawang B, Sangsawang N (2013). Stress urinary incontinence in pregnant women: a review of prevalence, pathophysiology, and treatment. Int Urogynecol J.

[2] Moossdorff-Steinhauser HF, Berghmans BC, Spaanderman ME, Bols EM (2021). Prevalence, incidence and bothersomeness of urinary incontinence in pregnancy: a systematic review and meta-analysis. Int Urogynecol J.

[3] Arrue Gabilondo M, Ginto L, Zubikarai M, Galán C, Saro J, Diez-Itza  I (2021). Risk factors associated with stress urinary incontinence 12 years after first delivery. Int Urogynecol J.

[4] Salmon VE, Hay-Smith EJ, Jarvie R, Dean S, Terry R, Frawley H, Oborn E, Bayliss SE, Bick D, Davenport C, MacArthur C, Pearson M (2020). Implementing pelvic floor muscle training in women's childbearing years: a critical interpretive synthesis of individual, professional, and service issues. Neurourol Urodyn.

[5] Pintos-Díaz MZ, Alonso-Blanco C, Parás-Bravo P, Fernández-de-Las-Peñas C, Paz-Zulueta M, Fradejas-Sastre V, Palacios-Ceña D (2019). Living with urinary incontinence: potential risks of women's health? A qualitative study on the perspectives of female patients seeking care for the first time in a specialized center. Int J Environ Res Public Health.

[6] Jaffar A, Mohd-Sidik S, Manaf RA, Foo C, Gan Q, Saad H (2021). Quality of life among pregnant women with urinary incontinence: a cross-sectional study in a Malaysian primary care clinic. PLoS One.

[7] Terry R, Jarvie R, Hay-Smith J, Salmon V, Pearson M, Boddy K, MacArthur C, Dean S (2020). "Are you doing your pelvic floor?" An ethnographic exploration of the interaction between women and midwives about pelvic floor muscle exercises (PFME) during pregnancy. Midwifery.

[8] Woodley SJ, Hay-Smith EJ (2021). Narrative review of pelvic floor muscle training for childbearing women-why, when, what, and how. Int Urogynecol J.

[9] Woodley SJ, Lawrenson P, Boyle R, Cody JD, Mørkved Siv, Kernohan A, Hay-Smith EJ (2020). Pelvic floor muscle training for preventing and treating urinary and faecal incontinence in antenatal and postnatal women. Cochrane Database Syst Rev.

[10] National Institute for Health and Care Excellence (NICE) (2021). Pelvic Floor Dysfunction: Prevention and Non-surgical Management [NG210].

[11] Lu J, Zhang H, Liu L, Jin W, Gao J, Min M, Fan Y (2021). Meta-analysis of perinatal pelvic floor muscle training on urinary incontinence. West J Nurs Res.

[12] Jaffar A, Mohd-Sidik S, Nien FC, Fu GQ, Talib NH (2020). Urinary incontinence and its association with pelvic floor muscle exercise among pregnant women attending a primary care clinic in Selangor, Malaysia. PLoS One.

[13] Grant A, Currie S (2020). Qualitative exploration of the acceptability of a postnatal pelvic floor muscle training intervention to prevent urinary incontinence. BMC Womens Health.

[14] Perera J, Kirthinanda DS, Wijeratne S, Wickramarachchi TK (2014). Descriptive cross sectional study on prevalence, perceptions, predisposing factors and health seeking behaviour of women with stress urinary incontinence. BMC Womens Health.

[15] van den Muijsenbergh ME, Lagro-Janssen TA (2006). Urinary incontinence in Moroccan and Turkish women: a qualitative study on impact and preferences for treatment. Br J Gen Pract.

[16] Asklund I, Samuelsson E, Hamberg K, Umefjord G, Sjöström M (2019). User experience of an app-based treatment for stress urinary incontinence: qualitative interview study. J Med Internet Res.

[17] Barton AJ (2012). The regulation of mobile health applications. BMC Med.

[18] Pires IM, Marques G, Garcia NM, Flórez-Revuelta F, Ponciano V, Oniani S (2020). A research on the classification and applicability of the mobile health applications. J Pers Med.

[19] Harjumaa M, Oinas-Kukkonen H (2007). Persuasion Theories and IT Design.

[20] Fogg BJ (2003). Persuasive Technology: Using Computers to Change What We ThinkDo.

[21] Oinas-Kukkonen H, Harjumaa M (2008). A Systematic Framework for Designing and Evaluating Persuasive Systems.

[22] Shao X, Oinas-Kukkonen H (2018). Thinking About Persuasive Technology from the Strategic Business Perspective: A Call for Research on Cost-Based Competitive Advantage.

[23] Orji R, Moffatt K (2018). Persuasive technology for health and wellness: state-of-the-art and emerging trends. Health Informatics J.

[24] Michie S, Fixsen D, Grimshaw JM, Eccles MP (2009). Specifying and reporting complex behaviour change interventions: the need for a scientific method. Implement Sci.

[25] Li T, Chen X, Wang J, Chen L, Cai W (2021). Mobile app-based intervention for pregnant women with stress urinary incontinence: protocol for a hybrid effectiveness-implementation trial. JMIR Res Protoc.

[26] Ammerman AS, Lindquist CH, Lohr KN, Hersey J (2002). The efficacy of behavioral interventions to modify dietary fat and fruit and vegetable intake: a review of the evidence. Prev Med.

[27] Richardson M, Khouja CL, Sutcliffe K, Thomas J (2019). Using the theoretical domains framework and the behavioural change wheel in an overarching synthesis of systematic reviews. BMJ Open.

[28] McClurg D, Frawley H, Hay-Smith J, Dean S, Chen S, Chiarelli P, Mair F, Dumoulin C (2015). Scoping review of adherence promotion theories in pelvic floor muscle training - 2011 ICS state-of-the-science seminar research paper i of iv. Neurourol Urodyn.

[29] Michie S, van Stralen MM, West R (2011). The behaviour change wheel: a new method for characterising and designing behaviour change interventions. Implement Sci.

[30] Danaher BG, Brendryen H, Seeley JR, Tyler MS, Woolley T (2015). From black box to toolbox: outlining device functionality, engagement activities, and the pervasive information architecture of mHealth interventions. Internet Interv.

[31] Chapman D (2009). Health-related databases. J Can Acad Child Adolesc Psychiatry.

[32] Higgins J, Thomas J, Chandler J, Cumpston M, Li T, Page M, Welch V (2019). Cochrane Handbook for Systematic Reviews of Interventions.

[33] Kim SY, Park JE, Lee YJ, Seo H, Sheen S, Hahn S, Jang B, Son H (2013). Testing a tool for assessing the risk of bias for nonrandomized studies showed moderate reliability and promising validity. J Clin Epidemiol.

[34] Araujo CC, Marques AD, Juliato CR (2020). The adherence of home pelvic floor muscles training using a mobile device application for women with urinary incontinence: a randomized controlled trial. Female Pelvic Med Reconstr Surg.

[35] Asklund I, Nyström E, Sjöström M, Umefjord G, Stenlund H, Samuelsson E (2017). Mobile app for treatment of stress urinary incontinence: a randomized controlled trial. Neurourol Urodyn.

[36] Dufour S, Fedorkow D, Kun J, Deng SX, Fang Q (2019). Exploring the impact of a mobile health solution for postpartum pelvic floor muscle training: pilot randomized controlled feasibility study. JMIR Mhealth Uhealth.

[37] Goode PS, Markland AD, Echt KV, Slay L, Barnacastle S, Hale G, Wright MK, Lane TR, Burgio KL (2020). A mobile telehealth program for behavioral treatment of urinary incontinence in women veterans: Development and pilot evaluation of MyHealtheBladder. Neurourol Urodyn.

[38] Han MN, Grisales T, Sridhar A (2019). Evaluation of a mobile application for pelvic floor exercises. Telemed J E Health.

[39] Hoffman V, Söderström L, Samuelsson E (2017). Self-management of stress urinary incontinence via a mobile app: two-year follow-up of a randomized controlled trial. Acta Obstet Gynecol Scand.

[40] Li J, Sun X, Wang C, Zhang Z, Xie Z (2020). A mobile application penyikang applied in postpartum pelvic floor dysfunction: a cross-sectional study to analyze the factors influencing postpartum pelvic floor muscle strength and women's participation in treatment. Biomed Res Int.

[41] Robson M (2017). The Squeezy pelvic floor muscle exercise app: user satisfaction survey. J Pelvic Obstet Gynaecol Physiother.

[42] Sjöström M, Lindholm L, Samuelsson E (2017). Mobile app for treatment of stress urinary incontinence: a cost-effectiveness analysis. J Med Internet Res.

[43] Wang X, Xu X, Luo J, Chen Z, Feng S (2020). Effect of app-based audio guidance pelvic floor muscle training on treatment of stress urinary incontinence in primiparas: a randomized controlled trial. Int J Nurs Stud.

[44] Bunzli S, Gillham D, Esterman A (2011). Physiotherapy-provided operant conditioning in the management of low back pain disability: a systematic review. Physiother Res Int.

[45] Hawkins RP, Kreuter M, Resnicow K, Fishbein M, Dijkstra A (2008). Understanding tailoring in communicating about health. Health Educ Res.

[46] Wadensten T, Nyström E, Franzén K, Lindam A, Wasteson E, Samuelsson E (2021). A mobile app for self-management of urgency and mixed urinary incontinence in women: randomized controlled trial. J Med Internet Res.

[47] Rygh P, Asklund I, Samuelsson E (2021). Real-world effectiveness of app-based treatment for urinary incontinence: a cohort study. BMJ Open.

[48] Jaffar A, Mohd Sidik S, Foo CN, Muhammad NA, Abdul Manaf R, Fadhilah Ismail SI, Suhaili N (2021). Protocol of a single-blind two-arm (Waitlist Control) parallel-group randomised controlled pilot feasibility study for mHealth app among incontinent pregnant women. Int J Environ Res Public Health.

