# Supplementary material for: Persuasive Technology in an mHealth App Designed for Pelvic Floor Muscle Training Among Women: Systematic Review
Source: JMIR Mhealth Uhealth. 2022 Mar 22;10(3):e28751. doi: 10.2196/28751 (PMC8984823; doi:10.2196/28751)
Supplement: Multimedia Appendix 1 [file mhealth_v10i3e28751_app1.pdf]

Figure S1, Multimedia Appendix 1: Quality assessment of included studies (RCTs)

	Random sequence generation (Selection bias)	Allocation concealment (Selection bias)	Blinding of participants and personnel (Performance bias)	Blinding of outcome assessments (Detection bias)	Incomplete outcome data (Attrition bias)	Selective reporting (Reporting bias)	Other bias
Asklund I et al., 2017	-	+	+	+	+	+	+
Hoffman V et al., 2017	-	+	+	+	+	+	+
Sjöström M et al., 2017	-	+	+	+	+	+	+
Araujo CC et al., 2019	-	+	+	+	+	+	+
Dufour S et al., 2019	+	+	+	+	+	+	+
Wang X et al., 2020	+	+	+	+	+	+	+

Note: The symbols "+", "-", and "?" indicate low, high, and unclear risk of bias, respectively

Figure S2, Multimedia Appendix 2: Quality assessment of included studies (Non-RCTs)

Citation	Study Design	Does the study have a strategy for minimizing selection bias, such as a comparison group?	Does the study use strategies to control for confounding, such as multivariable analyses and matching?	Is the study free of problems with how the exposure was measured (e.g. combining data with other types of mHealth app)?	Does the study have acceptable response rates (>50%) and/or attrition bias (e.g. dropout rate < 10%)	Is information provided about point estimates and errors?
Robson, M 2017	Cross-sectional, observational	No	No	Yes	No	No
Juan Li et al., 2020	Cross-sectional, observational	No	No	Yes	No	No
Michelle N. Han et al., 2019	Cross-sectional, observational	No	No	Yes	Yes	No
Patricia S. Goode et al., 2020	Pilot single group quasi-experimental study	No	No	Yes	No	Yes
